# A Qualitative Exploration of Parental Perceptions Regarding Children's Sun Exposure, Sun Protection, and Sunburn

**DOI:** 10.3389/fpubh.2021.596253

**Published:** 2021-02-18

**Authors:** Karlijn Thoonen, Liesbeth van Osch, Rowan Drittij, Hein de Vries, Francine Schneider

**Affiliations:** ^1^Department of Health Promotion, Care and Public Health Research Institute, Maastricht University, Maastricht, Netherlands; ^2^Faculty of Health, Medicine and Life Sciences, Health Sciences Master, Health Education and Promotion, Maastricht University, Maastricht, Netherlands

**Keywords:** sunburn, skin cancer prevention, sun protection behavior, children's health, parenting, health promotion, tanned skin, health education and behavior

## Abstract

Sun protection among children is of utmost importance since sunburn in early life is a major risk factor for skin cancer development. Because parents play a vital role in enhancing sun safety among children, this study explored parental perceptions concerning sun exposure, sun protection behaviors, and sunburn in children. Additionally, the context in which children experience sunburn in order to assist the development, optimization, and targeting of sun safety interventions for parents is revealed. A qualitative study design, using a semi-structured interview guide addressing several themes (e.g., sun exposure, sun protection, and sunburn experiences), was used. Data were collected in the Netherlands in the fall of 2019. Parents were recruited via purposive sampling at schools, youth services centers, and social media. Execution, transcription, and coding of the interviews was done by two researchers, using the qualitative analyzing program Nvivo (interrater reliability of *d* = 0.84). In total, 26 interviews were performed (*n* = 17 mothers, *n* = 17 daughters, aged between 4 and 11 years). Parental perceptions and recall of their child's lifetime sunburn were frequent, even though all parents reported using at least one sun protection measure during sun exposure situations and parents seemed often unaware of their child's sunburn. Moreover, parents reported an overreliance on sunscreen, often failing to adequately protect their children's skin. Water-related activities, a lack of shade, and misconceptions regarding UV-index were often related to sunburn. In addition, unexpected sun exposure or longer exposure duration than initially planned were reported as challenging situations. The majority of parents had positive perceptions regarding tanned skin for both themselves as for children. This study provides directions for skin cancer prevention efforts targeted at both parents and their children. Since a lack of knowledge regarding sufficient sun protection measures and sunburn occurrence in various situations was reported, educational efforts are warranted. Additionally, focusing on clothing, shade-seeking, and adequate sunscreen use is recommended to increase children's sun safety. By intervening in the physical environment as well (e.g., providing shady areas), sun protection barriers can be reduced. Lastly, the general positive attitude toward tanned skin evident in this study is certainly worthy of attention in future interventions.

## Introduction

Melanoma and non-melanoma skin cancers currently represent the most common types of cancer among fair-skinned populations, with exceedingly increasing incidence rates worldwide in recent decades (1–3). The lifetime risk of developing melanoma, the most fatal form of skin cancers, was estimated at 1 in 39 for men and 1 in 58 for women in the United States (4) compared to 1 in 16 and 1 in 24, respectively, in Australia (5). In Europe, the highest lifetime risk is observed in Nordic countries, ranging from 1.3 to 1.6% (6). Despite the relative stability of melanoma mortality rates across the United States, Australia, and Europe (1), management of skin cancers places a considerable and expanding burden on healthcare systems, and this is expected to worsen as skin cancer incidence increases (7). In the United States for example, the average yearly costs for the treatment of skin cancers rose by 126.2% between 2002 and 2011 (8). In Belgium, the economic burden of skin cancer was recently forecast to triple in the next two decades (9). Exposure to UVR (Ultraviolet radiation) and sunburn are considered major risk factors associated with melanoma development (3), where UVR-exposure and a history of one or more cases of sunburn during childhood are particularly harmful (10–12).

Although incidence rates have risen excessively in the past decades, skin cancers are considered to be one of the most preventable malignancies (13, 14). By protecting the skin and limiting the amount of unprotected exposure to UVR, skin cancer risk can be decreased. Various sun protection behaviors, such as avoiding the sun during peak UV-hours, wearing protective clothing, and applying a broad-spectrum sunscreen with SPF 15 or higher, are recommended strategies. Additionally, simultaneously practicing multiple sun protection methods is considered essential for adequate UVR-exposure protection, which accentuates the importance of primary prevention efforts (13, 15).

Despite the finding that sunburn incidence during early life profoundly increases the risk of melanoma (16), sunburn occurrence among children is highly prevalent, with recent percentages of children having experienced sunburn at least once in the previous year ranging from 28 to 60% (17–19). Besides the urgency to prevent sunburn during early childhood, establishing sun protection behaviors among children is especially advantageous since formation of health behavior patterns takes place in this critical period and such behavioral patterns are likely to persist into adulthood (20, 21). Parents and caregivers function as influential role models in the acquirement of children's own sun safety behaviors (22–24), which is notable throughout childhood. Young children generally depend on the direct protection behaviors that their parents apply (23, 25), whereas older children can indirectly learn to perform sun protection behaviors themselves through facilitation from their parents (26, 27). Although parents are recognized as crucial agents in teaching children health behaviors, a minority of sun safety interventions are directed at parents and those that are have limited effects on parental sun protection practices (25, 28, 29). Acquiring adequate sun safety practices among both children and their parents is therefore considered essential if future skin cancer rates are to decrease.

Although recommended strategies to increase sun safety are clear, inadequate sun protection during childhood is common (17, 30, 31). Suboptimal sun protection among children could be explained by weak intentions to execute sun protection behaviors. For example, poor intentions can be caused by low levels of knowledge (e.g., not knowing what sun protection measure is needed), attitude (e.g., not believing in the importance of sun protection), or self-efficacy (e.g., not feeling able to perform sun protection behaviors) (32, 33). Inadequate knowledge concerning the UV index and the necessity of sun protection measures (34–37), or experienced difficulty with performing sun protection behaviors (38, 39), can negatively affect behavioral outcomes. Moreover, studies have shown that parents' positive attitudes toward tanning can result in inadequate sun protection behaviors as well (30, 35). However, insufficient sun protection could also be explained by intentions not being translated into actual behavioral performance (40). For instance, perceived barriers or a lack of skills or action plans to perform sun protection can hinder sun protection intentions resulting in behavior (41). Barriers such as children's refusal of sun protection (42) or sun protection methods being perceived as impractical or unpleasant (43, 44) negatively affect sun protection behaviors. This intention-behavior gap has been previously demonstrated in studies in which parental intentions to apply sun safety measures did not result in actual sun protection behaviors (33, 45). Although intentions play a decisive role in establishing behavior change, it is important to recognize that not all behavior originates as a result of a reasoned approach but can also be non-deliberate and automatically generated by environmental cues (46). Several characteristics in the physical environment can influence whether or not behavior is performed [i.e., physical, economic, sociocultural, and economic (47, 48)]. Moreover, as the Environmental Research Framework for Weight Gain prevention (EnRG) states, different environmental levels of influence are distinguished as well, such as micro- (i.e., family, school) and macro- (government) settings (48). With regard to sun safety interventions, adaptations in the physical environment (e.g., provision of shady areas) can trigger sun protection behavior (49, 50). Especially since the dosage of received UVR by children is highly dependent on characteristics in the physical environment (51–53), insight into the environmental context affecting parental sun protection behaviors is needed.

Specific insight into situations in which children's sun safety is challenging and the risk of sunburn is elevated is lacking. Additionally, insight into contextual and situational factors besides motivational factors burdening children's sun safety is warranted to understand sunburn occurrence more clearly (54). Therefore, comprehending facilitating factors and barriers causing insufficient sun protection practices among parents in detail is needed.

In conclusion, insight into situational as well as environmental barriers hindering parental sun protection behavior needs elaboration. Moreover, in order to understand the challenges related to children's sun safety in view of those factors more clearly, exploration of parental perceptions regarding sun exposure and protection behaviors is necessary. This study therefore aims to unravel parental perceptions regarding their children's sun exposure, sun protection, and sunburn, in order to assist the development, optimization, and targeting of children's sun safety interventions directed at parents.

## Materials and Methods

A qualitative study design with semi-structured individual interviews was used, which enabled various themes to be addressed and a broad input from participants (55). Data collection took place between September and November 2019. The interviews were conducted individually and by telephone, since anonymity was considered highly important (56). Moreover, this approach enabled participants to remain in their private home setting, which was regarded most suitable given the possible sensitivity of some questions. Participants, and in particular parents, may be unwilling to acknowledge behaviors or circumstances that deviate from societal norms (57), especially when a child's well-being is under discussion, and parents can experience guilt or feel they are being blamed (58). Ethical approval for this study was granted by the Research and Ethics committee of the Faculty of Health, Medicine and Life Sciences of Maastricht University, under the general license of the Master Health Education and Promotion. Verbal informed consent of all parents was obtained and recorded. This study is reported in accordance with the COREQ checklist for qualitative research (59).

### Participants and Recruitment

**Figure 1:**
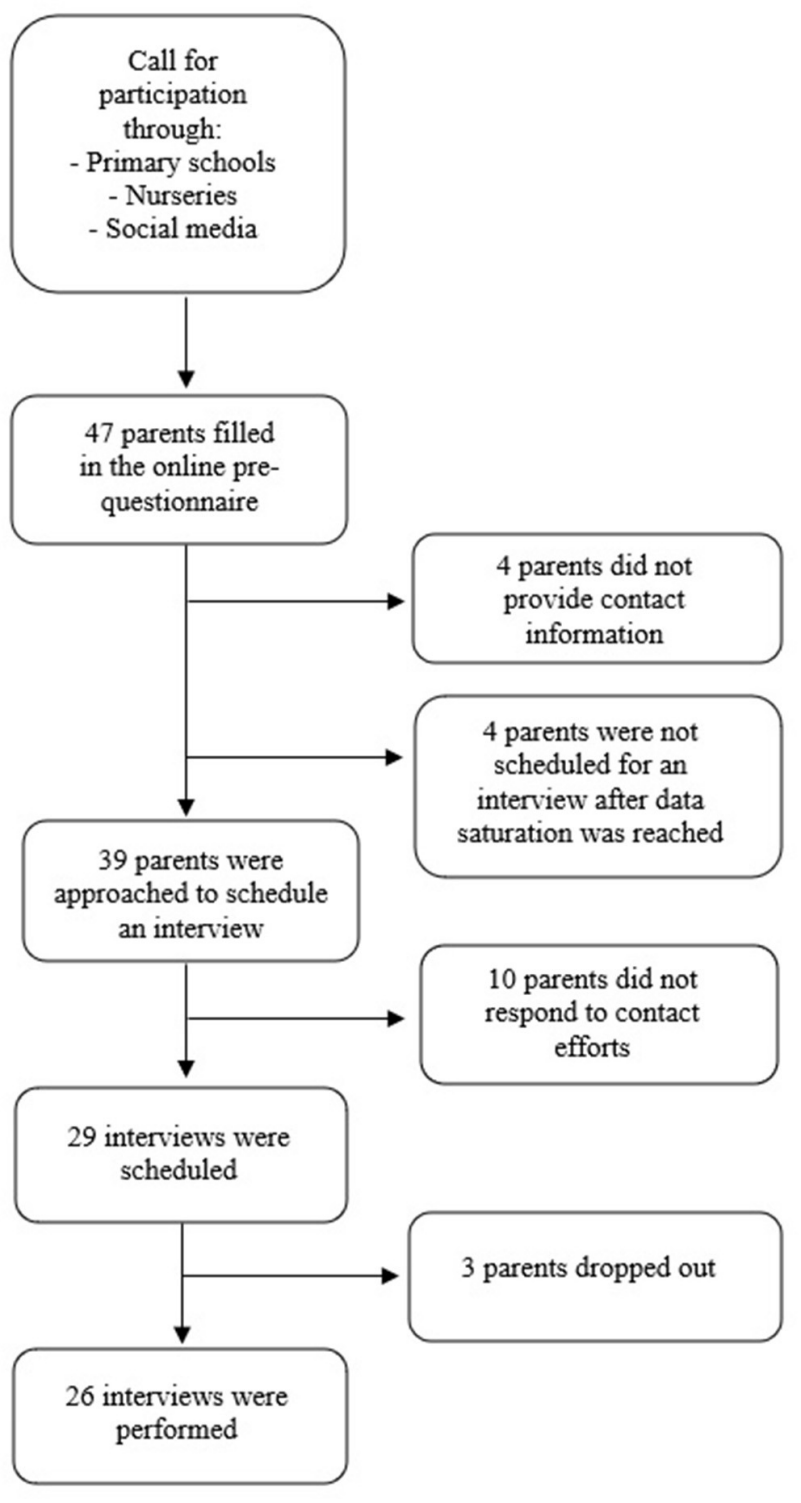
Flowchart of participants' recruitment.

Parents with at least one child aged between 4 and 12 and speaking either Dutch or English were eligible for inclusion in this study. A flier was created in which parents were requested to participate in an interview to share information about their children's sun exposure during the previous summer season. The flier was disseminated via several recruitment channels; primary schools and a childcare center were approached, after which social media sites were used. These channels were carefully chosen since they were expected to recruit information-rich cases for this study (60). Firstly, three primary school boards in the east of the Netherlands, and one large daycare center in the south were approached by e-mail, explaining the purpose of the study and asking them to distribute an online recruitment flier. Secondly, social media platforms were used for further recruitment. Facebook was used to gain access to various pages and groups specifically directed at parents (e.g., pages containing education and information about parenting, health center pages for children and families, and youth healthcare organizations). Permission for recruiting participants by posting an online flier was requested by directly contacting the group moderators online. See Figure 1 for a flowchart of the recruitment process.

### Procedure

Parents could indicate their interest in participating in this study by clicking on a link provided in the recruitment flier, after which they were directed to a survey website [Qualtrics, Provo, UT (61)]. The website specified the aim of the study, including gaining information concerning children's sun exposure and sun protection during the previous summer. Research members' names and professions were also mentioned. On the website, parents were asked to fill in the age of their children as a check for eligibility criteria. Other demographic questions (i.e., parental sex, educational level, children's sex) were also assessed. Finally, parents were asked to indicate whether they were willing to allow the researchers to approach them via telephone and e-mail to schedule a telephonic interview by providing their contact information. The researchers selected parents using a purposive sampling strategy (62), striving for an equal distribution of demographic characteristics of both parents and their children since differences in terms of demographic variables can affect health-related outcomes (63). An equal as possible distribution of children's ages was the primary goal and a representativeness of children's sex the secondary goal when approaching interested parents for the interviews. With regard to children's age, a representation of children from all three phases of the Dutch primary school system was strived for (4 to 6 years, 7 to 9 years, and >10 year). Children's skin type was unknown before scheduling the interviews. Aside from the demographic characteristics, the researchers were not familiar nor affiliated with the participants in any way. When approaching the parents, further information regarding the study aim was provided and telephonic interviews were scheduled. Parents then received a confirmation e-mail along with an information letter containing data collection procedures, recordings of the interviews, storage of data, participant's protection of anonymity, and their rights to withdraw from the study. After completing the interviews, participants received an incentive of a 10-euro gift voucher, which was sent to their home address. To increase reliability and validity, member checking was done by paraphrasing and summarizing the information provided throughout the interviews (64). Participants did not receive the verbatim transcripts. After concluding the study, an overview of study results, accompanied by an infographic including sun protection information and tips, was sent to all participants. Both researchers had a professional background in patient communication in a healthcare setting. A semi-structured interview guide was used to structure the conversations.

To accomplish both investigator and method triangulation, the interviews were alternately conducted by one of the two researchers, while the other researcher present generated interview notes based on observations (65). Before commencing the interviews, audiotape devices were switched on and the interviewer asked whether participants had read the study information letter and whether they had any questions regarding the information. If participants had not read the information, the interviewer outlined the most important study aspects as mentioned above and asked whether participants had any questions. The interviewer then read out loud a prepared text in which the study purpose, data collection and data storage methods, and participant's rights from the previously sent information letter were summarized, and verbal informed consent was obtained. Thereafter, parents were asked to answer all questions for one specific child. Before the interview continued, the researcher explained the purpose of the interview and told participants that they were being asked about their personal experience regarding all the themes, and emphasized that there were no right or wrong answers. Each interview lasted ~30 min. To increase reliability and validity, the participant's information was summarized and paraphrased regularly throughout the interviews (64). As well as interview guides, a field note format was used in which the overall impression of the conversation as well as practical issues, critical reflections of the researcher's performance, and possible biases were observed and reported (66). Interviews were scheduled and conducted until sufficient information was obtained and data saturation was reached (67), enabling the researchers to proceed with the data analysis process.

### Interview Guide

Prior to the interviews, a semi-structured interview guide was developed, consisting of three main themes, namely, perceptions regarding: (1) sun exposure; (2) sun protection behaviors; and (3) sunburn. Additionally, parental and children's skin type were assessed and estimations of children's skin type were later formulated by the researchers based on the Fitzpatrick skin type classification (68). Existing literature concerning sun protection behaviors as well as concepts from the environmental EnRG framework (48) were used to frame the three themes. The interview guide was reviewed by the whole research team on language use and content, and was adjusted where necessary. To test the suitability and feasibility of the interview guide, three interviews were piloted. The research team discussed the preliminary results of the interviews before making slight adaptations to the questions and their sequence.

The first two themes of the interview guide consisted of questions regarding negative and positive aspects of sun exposure. These parts were preceded by an open-ended question about perceptions that parents held regarding the sun, after which prompting questions were asked to retrieve in-depth information about three domains, i.e., (1) health-related; (2) well-being (mental health); and (3) appearance-related. The third part of the interview guide entailed questions regarding direct parental sun protection behaviors, children's own sun protection behaviors as well as indirect parental protection behaviors (i.e., facilitating their child). This part was introduced by explaining that different measures can be taken to protect one's skin against the sun. In contrast to the previous parts of the interview, the questions regarding sun protection behaviors were more structured.

When participants were not very forthcoming, the researcher included prompt questions regarding the three main domains (55). In the first part, independent of whether participants mentioned it themselves, the researcher mentioned sunburn occurrence as a possible negative aspect of the sun. Then, the researcher asked whether the child had experienced sunburn in the previous summer season, after which the researcher asked detailed questions about this specific situation. If parents did not report a previous case of sunburn, the researcher asked them whether they could recall details from an earlier sunburn situation in their child's life. When parents stated that no sunburn had ever occurred, parents were asked to imagine a potential sunburn situation. Further in-depth questions regarding the characteristics of experienced or imagined sunburn situations were then posed. In the second part, again independent of participants mentioning it themselves, a tanned skin was introduced. Thereafter, in-depth questions were asked concerning parental perceptions as well as other people's opinions regarding children's tanned skin. The themes from the interview guide with exemplary items are depicted in Table 1.

**Table 1:** Interview themes and exemplary items.

**Theme**	**Category**	**Exemplary Items**
**Negative aspects of the sun**		
	Open associations	“*What comes to mind when you think about disadvantages of being in the sun?”*
	Prompted associations	“*What possible disadvantages of the sun are there regarding emotions or how you feel?”*
	Sunburn situation	“*What was the purpose of the activity?”* *“Was there a lot of cloud coverage?”* *“How severe was the sunburn?”* *“What made sun protection in this situation particularly difficult?”*
**Positive aspects of the sun**		
	Open associations	‘*What comes to mind when you think about benefits of being in the sun?'*
	Prompted associations	‘*What possible benefits of the sun are there regarding your health?'*
	A tanned skin	“*How important is a tanned skin for you? And how important is a tanned skin for you when it concerns your child?”* *“How do you feel about your child having a tanned skin?”* *“What do you think other people around you feel about your child having a tanned skin?”*
**Sun protection behaviors**		
	Parental behaviors	Direct: “*Which sun protection measures do you apply when your child is exposed to the sun?”* Indirect: “*To what extent do you support your child in executing sun protection behaviors him- or herself?”*
	Children's behaviors	“*Which sun protection methods does your child apply him- or herself?”* “*What would facilitate you or your child to perform sun protection measures in the future?”*

### Data Analysis

After recording the interviews, verbatim transcripts were generated and imputed in the qualitative analyzing program NVivo, version 12 (69). In accordance with the six phases of thematic analysis (70), first, each researcher individually open-coded one different and randomly selected transcript and developed a codebook. Then, the two codebooks were compared, and a new codebook was composed. The researchers then used this codebook during their independent coding of the same selection of five transcripts. Hereafter, an interrater reliability was calculated in NVivo, based on Cohen's Kappa, resulting in a high level of agreement (*k* = 0.84) between the two coding results (71, 72). Lastly, an adjusted version of the codebook was generated by mutual agreement after discussing small disparities by comparing coded passages. The finalized codebook was used to code the remaining transcripts and to formulate main themes and categories to enable the results to be interpreted. A third researcher (FS) observed and reflected on the analyzing process to enhance objectivity. Thematic coding took place by clustering codes together in categories, followed by interpretation of these categories and their integration into several main themes. Again, discrepancies were discussed by comparing passages and mutually adjusting the categories.

## Results

### Sample Characteristics

Forty-seven participants filled out the online questionnaire, of which forty-three parents submitted their contact information for scheduling an appointment. Thirty-nine parents were approached throughout the study period and an interview was scheduled with twenty-nine parents, based on a previously made selection according to children's and parental age, children's and parental sex, and educational level. Four interested parents were not scheduled for an interview after data saturation was reached. Three parents dropped out before the interview took place, due to being inaccessible (*n* = 2, mothers) and health-related problems (*n* = 1, father). The dropped-out parents all had a son (aged 6, 9, and 10). In total, twenty-six telephonic interviews with parents (17 mothers, 9 fathers) of children (17 daughters, 9 sons) aged 4 to 11 years (M = 7 years, Modi = 4, 5, and 7 years) were conducted. The age of the parents ranged from 30 to 50 years (M = 40 years, Modus = 44) and the majority of the parents were highly educated (*n* = 20; 76.9%). Most children had a very light (*n* = 6) or light (*n* = 8) skin type, and one child had a darker skin type based on the Fitzpatrick skin type classification. Almost all parents (*N* = 25; 96%) reported that their child had experienced sunburn at least once in their lifetime. An overview of emerged themes and sub-codes is attached as a Supplementary Table 1.

### Perceptions Regarding Sun Exposure

#### Negative and Positive Perceptions

Parents did not immediately mention general negative perceptions regarding sun exposure. Consequently, pro-active prompts by the interviewers regarding disadvantages were necessary. After prompting, negative aspects of the sun in general that were subsequently mentioned were most frequently health- and appearance-related. The risk of sunburn and of developing skin cancer was mentioned most often: “*… Yes, too much UV-radiation, you read it everywhere nowadays, that, that it can cause skin cancer, ehm, yes, that is obviously the greatest disadvantage of UV-radiation.”* Additionally, damaged skin, dehydration, heat stress, and getting sunstroke were indicated. After prompting, by asking about possible appearance-related disadvantages, parents perceived getting a wrinkled skin, freckles, and (hyper) pigmentation or spots as being general negative aspects of the sun. With regard to negative aspects of the sun on well-being, parents indicated the heat from sunrays was sometimes excessive.

On the other hand, parents often mentioned particularly perceiving the sun as yielding positive rather than negative aspects, and extensive lists of benefits were reported throughout all interviews. Positive perceptions mentioned were health-, well-being-, and appearance-related. Health-related perceptions reported were the sun being the reason to be outside more and children being more physically active, fit, living a healthy life, and receiving sufficient vitamin D: “*And for me, I really get energy by sitting in the sun.”* Parents perceived being in the sun as considerably important and part of everyday life. Mental well-being was perceived evidently and positively affected by the sun, as parents observed an improved mood for themselves as well as their children when being in the sun. Several aspects, like feeling happy and joyous, energetic, being more socially active, and enjoying the warmth on the skin, were mentioned: “*. it is also just a general feeling of happiness.”*

#### Perceptions Regarding a Tanned Skin

One theme that frequently emerged without prompting, was appearance-related. The majority of parents perceived a tanned skin, resulting from sun exposure, as being positive, pretty, and more pleasant and healthier than pale skin, in both adults and children: “*In my experience, I think people* (with a tanned skin) *look more fit, more alive, yes, healthier,”* and a pale skin was often associated with illness, being unhealthy, or a lack of nutrition. Interestingly, one parent particularly mentioned a pale skin as being prettier than a tanned skin: “*I think that it* (pale skin) *is pretty. Prettier, yes, I really like it better than a tanned and wrinkled skin.”* The positive perceptions regarding a tanned skin are often reinforced by friends or family members complimenting parents or their children on the way it looks, especially after a holiday. Additionally, a tanned skin was not perceived as positive when it was the result of sunbed use, with some parents using the term “*sunbed-tanned”* to explain their negative perceptions accordingly, along with being unhealthy, tacky, or not attractive. Moreover, parents considered the extent to which a skin is tanned as being important: “*It depends on the gradation; if it becomes like a super-tanned skin, I don't think that that's healthy at all.”* Despite the positive perceptions, parents stated that they did not intentionally strive after a tanned skin for their children and a tan often occurred by chance. However, several statements of parents indicated that their preference for a tanned skin may play a role in performing sun protection measures for their child: “*… though I'm probably thinking about it unconsciously, since I kind of like a bit of a tan,”* and “*… deep in my heart I think it looks nice. But then I think oh, uh, I only rub them in with sunscreen at noon and not in the morning for example. It* (leaving the child unprotected in the sun before noon) *is possible*.”

### Perceptions Regarding Sun Protection

All parents perceived sun protection as being important, were aware and well-informed about different sun protection measures, and performed both direct and indirect sun protection behaviors. Furthermore, mothers most often facilitated sun protection measures in advance of a situation (e.g., buying sunscreen, bringing sunglasses), whereas fathers were often initiators of sun protection during sun exposure situations. Nonetheless, all parents in a relationship perceived their parental role in their children's sun protection of equal importance and regarded it as a result of teamwork with their spouse. Motivational factors for parental sun protection performance were most often a light (or lighter) skin type, a history of sunburn, and knowing people who had had, or still had, a form of skin cancer.

#### Direct Sun Protection Behaviors

Parents applied sunscreen, with SPF50, often as a singular and predominant method, even though they were well-aware of the importance of sun avoidance and wearing protective clothing: “*Yes, no, it's not that I take clothing into account. No, I mean, it is adequate to use sunscreen if you apply it sufficiently (…). Well, you could think of wearing sunglasses and that sort of thing… But principally it is sunscreen.”* However, parents perceived a large number of barriers concerning sunscreen application, such as ambiguity about the working mechanisms of sunscreen (e.g., protection duration, recommended amount of sunscreen, or whether sunscreen is waterproof):“*… I don't know, did I have to reapply* (the sunscreen) *every 30 min instead of every hour? For me, this is very difficult,”* or “*Yes, if I can be absolutely sure that it* (water-resistant sunscreen) *would work effectively for 2–3 h, I would definitely buy it.”*

Shade-seeking behavior as an additive to sunscreen was the second most practiced behavior, with parents indicating using trees, parasols, beach tents, or staying inside as protection measures for their children. Although specifying that seeking shade was important, parents often only sought shade when practicing a particular activity, such as eating or resting, or after their children's skin had already turned red. Parents perceived barriers with sun avoidance since shady areas were often not available at venues where their children were playing: “*…there is a chance that there is not any shade at all* (when being outside). *And it's uhm, for hours and hours.”*

Even though putting on clothes was performed the least, some parents mentioned (buying) UV-protective clothing specifically, without prompting. “*I once bought a UV-protective swimsuit or something like that* (…)*, but it remains in the closet with the price tag still on it.”* Several parents indicated the use of T-shirts on very sunny days, although clothing was considered as too hot and uncomfortable for children, especially when they were playing near the waterfront, and it being a hassle and impractical for parents: “*.Maybe that thought is wrong, but yeah, the children also don't like wearing long-sleeved clothing….”* However, wearing hats was frequently mentioned among younger children. Although sunglasses were rarely used, some parents mentioned that their child liked wearing sunglasses since “*… her mom and dad wear them too….”*

#### Indirect Sun Protection Behaviors

All parents performed indirect sun protection by stimulating or supporting their children to execute sun protection themselves, such as informing children about the importance of sun protection, demonstrating sun protection behaviors (e.g., by roleplaying with dolls), and actively teaching children to perform sun protection. The children's age at which parents started supporting children in practicing sun protection themselves varied widely (e.g., roleplaying at the age of 5 or putting sunscreen in the schoolbag from the age of 10). None of the parents had thought about future directions or plans concerning teaching their children sun protection behaviors: “*Oh, well, I haven't made any* (future) *plans to be honest* (laughing).”

Although parents played an important role in children's sun safety, most children were, to a certain extent, able to perform sun protection behaviors themselves as well. The age at which children started executing behaviors themselves varied widely, with e.g., children starting to apply sunscreen or putting on sunglasses themselves ranging between 5 and 12 years old. Where some young children were independently applying sunscreen (7 years: “*…meanwhile they do it themselves, and if I forgot it, they would bring sunscreen to me, for example, and then say ‘we still have to do this'…”*), some older children did not perform sun protection themselves (11 years: “*No, we usually still do that. He is not very active in that area”*). No clear distinction between boys and girls was apparent, although some parents perceived girls as initiating sun protection from a younger age than boys. Overall, parents indicated that most children enjoyed performing sun protection behaviors themselves as it made them feel grown-up, cool, and comfortable: “*We put the sunscreen in her backpack and then she really uses it. She is really trying her best and likes doing it.”* Furthermore, parents preferring to be in control of their children's sun protection often performed all sun protection behaviors until their child was older, whereas parents specifying their children's own responsibility stimulated children's own sun protection from an earlier age: “*Well, you can't do much about it, right? So I hope she learns from us that it is important to use sunscreen and that she thinks of it, and uses it, herself* (laughing).”

### Perceptions Regarding Sunburn

Although all parents mentioned taking sunburn precautions, almost all children (96%) had experienced at least one case of sunburn in their lifetime: “*…the sun shines and then… she comes inside with especially red, really red cheeks, the cheeks are a bit discolored….”* Although not specifically asked for, twelve parents reported sunburn in the previous summer months. A few parents explicitly mentioned having been sunburnt themselves during their childhood and definitely not wanting their child to suffer as well: “*I mean, at very short notice it could be really, really painful right? I got really badly sunburnt myself and it's painful.”* Parents described sunburn situations either explicitly or implicitly, according to their recall of experienced or imagined sunburn. Additionally, the distinction between sun exposure being either anticipated or unanticipated in the occurrence of sunburn was revealed as being important.

#### Explicit and Implicit Sunburn Situations

Eighteen parents mentioned that they were able to explicitly recall one or more situations in which their child experienced sunburn. All of these parents reported feeling guilty and shocked as soon as they noticed the sunburn: “*… But I didn't pay enough attention and therefore she was suffering from it for the next few days and I felt sorry for her.”* When asked about the severity of children's sunburn, it appeared difficult for parents to define the sunburn: “*Hmm, not really burnt. I didn't think she was, that she was suffering from it. No painful sunburn. But I thought she was a bit too red on her calves…”* or “*Because sometimes shade is not enough to prevent sunburn. Well sunburn, at least deeper tanning….”* In some cases, parents initially stated that no sunburn had occurred, whereas after introducing one or two prompts, they did recall a sunburn situation. Parents mentioned water-related situations being a great challenge for various reasons, such as the water rinsing off sunscreen, children playing on the waterfront and refusing appliance or re-appliance, or characteristics in the physical environment impeding sun protection (e.g., absence of shade). Several parents mentioned the temperature as an indicator for necessary sun protection measures: “*But in the Netherlands I often think: ah well, it's not that bad at 23 degrees* (Celsius)…*,”* or: “*… and if it's very hot sometimes, applying sunscreen only once may not be sufficient ….”* The amount of cloudiness was also mentioned as making it difficult to estimate the UVR-strength: “*If it's a bit cloudy for example, you don't feel like ‘Oh, let's put on a hat', while the sun can still be very strong. That makes it so difficult.”*

Seven parents could either not recall their child having been sunburnt, or indicated that their child had never experienced sunburn, after which the researchers asked the parents to imagine a sunburn situation. Nonetheless, these parents were able to provide detailed information and examples regarding several potential sunburn situations and related barriers. The researchers identified these situations as implicit sunburn situations. Moreover, the observation emerged that these parents did not recognize sunburn as such, even though previously provided information proved otherwise, as illustrated by the following examples: “*… somewhat reddened skin, but not really burnt with blisters or other distress, so not really burnt, no,”* and “*Because, yes, it was red, but fortunately not burnt.”* These parents stated that they imagined it would be challenging not being present to have control over sun protection (e.g., their child being at school or at a friend's house), since teachers or other caretakers could be unaware about adequate sun protection measures. Second, situations in which children were engaging in outdoor activities (e.g., playing, biking, or at sports clubs), were considered to be difficult since the focus of the situation simply did not concern sun protection. Third, parents acknowledged several barriers related to the climate or the physical environment, such as wind and cloud coverage causing misinterpretation of the UV index.

#### Anticipated and Unanticipated Situations

When parents expected to be out in the sun, the majority took precautions by applying at least one sun protection method to protect their children's skin. Despite this, parents frequently mentioned being surprised by the strength of the sun, resulting in sunburn. Parents described feeling that their child's sunburn occurred suddenly, and that they were shocked by the rapidity of the event: “*Yes, it went faster than I thought. Maybe the power of the sun* (UV index) *was too strong and I think I underestimated that.”* During this expected sun exposure, parents were not able to intervene earlier since the critical point in time was too abrupt: “*…It took me by surprise. It* (sunburn) *went faster than I'd expected.”*

Furthermore, unexpected sun exposure occurred especially when the sun exposure lasted longer than anticipated or prepared for: “*Uhm but yes, sometimes they are having so much fun … and then uh, yes, then you sometimes forget that* (initiating sun protection).” In some cases, parents did not know their child was outside at all. When sun exposure was unexpected or not anticipated, parents did not bring sufficient sun protection products with them or their children were not shielded by protective clothing. Moreover, parents mentioned certain situations as not being associated with sun protection, such as when children engage in activities outside parental supervision when going to a friend's house or school: “*…and then you're walking to school and you suddenly remember: oops, we forgot to use sunscreen.”* Parents believed that having sun protection materials ready at all times could have helped them to prevent future cases of sunburn: “*Actually, having a backpack ready for sunny days, yes, and also with sunscreen in it, yes. And we should take that bag with us.”*

#### Facilitators

Besides barriers affecting adequate children's sun protection, parents also mentioned facilitating factors. With regard to *health education*, parents mentioned that increasing societal awareness regarding skin cancer risk and sun safety policy at schools and communities (e.g., sports clubs) would facilitate children's sun protection. Moreover, receiving up-to-date alerts regarding the current UV index via (online) weather forecasts, mobile apps, or on signs at recreational or public venues were mentioned. Almost all parents mentioned that they were in need of information about sufficient sun protection methods. Besides information targeted directly at them, parents felt that media campaigns could be helpful as well. Some parents believed that detailed information regarding their children's sun safety would have been helpful when their children were much younger (e.g., at the child health clinic or at daycare centers). Including children themselves in educational efforts was also described as facilitating.

Regarding *health promotion*, almost all parents mentioned shade availability at public venues as an important facilitator for sun protection behavior, along with signs reminding them to take sun protection measures. Moreover, since parents were often insecure about sufficient sun protection at school, shady areas in school playgrounds were often mentioned. Some parents also mentioned provision of sunscreen at public venues, such as at restaurants: “*…make sure that at restaurants a bottle of, eh, SPF50, is available. So that anyone can use it.”*

## Discussion

This study provides a comprehensive exploration of parental perceptions regarding sun exposure, sun protection, and sunburn. Additionally, situations in which children experienced sunburn revealed in-depth information, presenting potential directions for future prevention efforts.

Overall, almost all children, of which seven parents did not explicitly report one, had experienced at least one case of sunburn in their lifetime. The high prevalence of children's sunburn occurred although all parents reported their intentions to perform sun protection behaviors, as well as the use of at least one sun protection measure. Moreover, the application of sunscreen was the most frequently reported protection measure. The preference for sunscreen in this sample is in accordance with existing literature (26, 73), although almost all parents reported difficulties in adequate application and regarded sunscreen use as complex. Other sun protection behaviors (e.g., shade-seeking or wearing clothes) were executed less often, with parents perceiving them as even more challenging, which has also been reported previously (39, 74). The most frequently mentioned situations in which sunburn occurred were situations involving water, a lack of shade, cloudiness and low temperatures causing underestimation of the UV index, and being caught by surprise.

There were positive perceptions regarding a tanned skin, for both adults and children, among a majority of the parents, as well as in their social environment. The influence of desiring a tanned skin can affect sun protection choices (75, 76), with earlier studies revealing that parents with positive attitudes toward tanning tend to protect their children less adequately (24, 35, 38). This was not apparent in this study. Nonetheless, further investigation concerning the extent to which these attitudes affect sun protection behaviors is necessary. More importantly, the presence of normative beliefs approving and complimenting a tanned skin should be changed, since social norms have been found to influence parental sun protection behaviors as well (42, 77). In any case, modifying parental attitudes toward tanning in order to improve parental sun protection behaviors is highly recommended.

Although parents in this study were knowledgeable about general skin cancer risk, and motivated to perform sun protection behaviors, misconceptions concerning sun safety were still frequently detected. First, parents reported a reliance on sunscreen as sole prevention method, which could potentially explain the high prevalence of sunburn found in this study (78–81). Moreover, parents felt insecure and experienced ambiguity in the correct use of sunscreen. Sun safety recommendations emphasize the use of multiple sun protection methods (82). Since the lack of clear and consistent communication regarding sufficient sunscreen use has been drawn attention to earlier (13), educating parents about the necessity for adequately performing multiple sun protection behaviors is warranted. Second, parents often reported inaccurate estimations of the temperature and UV index, resulting in inadequate preparations for sun exposure situations. The confusion and lack of awareness concerning the relation between UV index, cloud coverage, and the need for sun protection measures among parents has been previously described (35, 36). Specifically, since parents were not always well-prepared for these weather conditions, awareness of the increased risk of sunburn in specific weather conditions is needed. Lastly, with regard to parental self-efficacy to perform sun protection, this study revealed that parents felt insecure, and perceived performing sun protection behaviors correctly as difficult. It is known that experiencing difficulties in performing sun protection behaviors, and low levels of self-efficacy, influence parental sun protection behaviors negatively (33, 38, 83). Further research to identify specific difficulties and barriers regarding sun protection is recommended in order to design sun safety interventions and teach parents adequate (coping) skills (84).

Second, characteristics in the physical environment, such as situations in which limited shade was available, and especially at public venues involving water, were perceived as particularly burdensome in adequately protecting children (22). Since the availability of shady areas increases the likelihood of shade-seeking behavior (49, 50), the importance of shade at recreational areas should be recognized. Moreover, warning signs involving the current UV index, along with specific advice for sun protection measures at public venues, could be beneficial.

Although some parents indicated that their children were, to some extent, able to perform sun protection measures themselves, none of the parents reported future plans for teaching their children sun protection behaviors. Previous studies revealed that children are able to enhance their own sun protection behaviors (26) and that attitudes in favor of sun protection decline when children become older (23, 85). Stimulating parents to teach their children to adequately practice sun protection behaviors themselves is crucial in order to increase the establishment of sun protection throughout life (20, 21).

While the high number of reported cases of sunburn may demonstrate that parents were willing to talk straightforwardly and honestly about their experiences, most parents had initial difficulties in mentioning sunburn explicitly. For example, at first, several parents indicated that either no sunburn had occurred after which they elaborately mentioned explicit factors causing sunburn. In addition, some seemed to minimize the severity of their children's sunburn or had difficulty distinguishing “deep tanning” from sunburn. On the one hand, this could potentially be explained by a tendency to provide socially desirable responding (86) and therefore attenuating the severity of sunburn. Parents mentioned feeling guilty and shocked when their child experienced sunburn, a finding congruent with a recent study (43). On the other hand, the initial underreporting of sunburn could be explained by parents not recognizing the classification of sunburn as such. The validity and reliability of self-reported sunburn have shown to be a great challenge, as illustrated in earlier work (87), although not specifically among parents. Hence, further exploration concerning this crucial topic is warranted to decide upon future intervention development. Furthermore, parents indicated that previous cases of sunburn among themselves and their children caused them to enhance their sun protection behaviors, which is in line with previously reported findings (43, 80). Nevertheless, contrasting findings in which previous cases of sunburn do not serve as potential cues to action for future sun protection behavior are known as well (38, 86, 88). Therefore, the influence of actual experience of sunburn in future sun protection behavior needs further investigation.

## Strengths and Limitations

This study has various strengths. To the best of our knowledge, this study is the first to explore parental perceptions regarding sun exposure, sun protection behavior, and sunburn among Dutch parents. The study provides an essential exploration that can guide future quantitative research and intervention development. The qualitative origin of this study enabled a broad understanding of parental perceptions regarding various themes. Since semi-structured interview guides were used (89), respondents provided elaborate input. This resulted in comprehensive information regarding extensive themes (e.g., parents' positive attitude toward tanning). Second, efforts were made to enhance heterogeneity in the sample of respondents. Although representativeness and generalization of samples used in qualitative studies is not possible (90), the influence of certain demographic characteristics (i.e., parental sex, age, and educational level, and children's sex and age) on sun protection behavior was acknowledged by striving for inclusion of parents with different backgrounds (62, 63). Third, to optimize the validity and reliability of this study, various strategies were applied. Multiple researchers worked closely together throughout the process, standardized topic guides and observation notes were used to minimize potential biases (66), and all interviews were critically reflected upon. Moreover, cross-checking of coding and calculating the inter-coder reliability were performed, showing an almost perfect level of agreement (71). Fourth, the chances of a social desirability bias may have been diminished by performing the interviews by telephone, rather than face to face, especially given the sensitivity of some questions (91).

Although this study has several strengths, a few limitations should be mentioned as well. First, with regard to participant selection, a response and selection bias could have occurred (62), resulting in a decreased representativeness of the sample. Since parents had to actively express interest in participation, demonstrated high levels of sun safety awareness, and were in general highly educated, sun protection behaviors and sunburn patterns may be different than those of less motivated and lower educated parents, as revealed in a study in which children of lower educated parents were less protected and more frequently sunburnt than those of higher educated parents (92). Moreover, the relatively low retention rate could have induced selection of motivated parents. Second, although the telephone interviews could have diminished possible social desirability, this bias cannot be ruled out (93). As illustrated in the results of this study, several parents initially indicated that their child had not suffered sunburn, as the interview progressed, the opposite turned out to be the case. The effects of a possible social desirability bias are expected to be minimal, given the high prevalence of reported sunburn in this study. Third, execution of parental sun protection behaviors was assessed using subjective, rather than objective measures. Together with a social desirability bias, the sensitivity of the themes could have induced a parental overestimation of their sun protection behaviors, as is also suggested in earlier work (94, 95). To validate a potential over reporting among parents, studies comparing self-reported and objectively measured sun protection could be beneficial (96).

## Conclusions

Educating parents about adequate preparation of sun exposure situations as well as specific weather- and environmental conditions should be considered in future sun safety interventions. Additionally, given its limited use among the parents in this study, a focus on wearing UV-protective clothing and other garments is also worthy of attention. Furthermore, a focus on environmental cues encouraging sun protection behaviors, such as increasing the availability of shady areas or warning signs concerning the UV index in the physical environment is recommended. Moreover, future research is recommended to investigate whether underreporting or underestimation of sunburn that appeared in this study is a result of either parental hesitation to admit to a child's sunburn or misconceptions about classifying sunburn. In any case, educational efforts to raise awareness and acknowledgment concerning the UV index, sunburn occurrence and severity, importance of simultaneously performing multiple sun protection behaviors, and focusing on teaching parents skills to overcome barriers, are warranted to enhance parental and children's sun safety. Additionally, reducing the desire to have a tanned skin among both parents and children needs further consideration.

## Data Availability Statement

The raw data supporting the conclusions of this article will be made available by the authors, without undue reservation.

## Author Contributions

KT, RD, LO, HV, and FS conceived and designed the study. KT and RD collected and analyzed data. FS supervised the study. KT, RD, and FS interpreted results. KT wrote the manuscript. All authors read and approved the final version of the manuscript.

## Conflict of Interest

The authors declare that the research was conducted in the absence of any commercial or financial relationships that could be construed as a potential conflict of interest.
