# Supplementary material for: A Qualitative Exploration of Parental Perceptions Regarding Children's Sun Exposure, Sun Protection, and Sunburn
Source: Front Public Health. 2021 Feb 18;9:596253. doi: 10.3389/fpubh.2021.596253 (PMC7930008; doi:10.3389/fpubh.2021.596253)
Supplement: Supplementary file 1 [file Table_1.docx]

| **Supplementary Table. Themes, codes and sub-codes** | | |
| --- | --- | --- |
| **Themes** | **Codes** | **Sub-codes** |
| **1. Sun exposure** |  |  |
| ***Negative perceptions*** | Health | Sunburn |
|  |  | Skin cancer |
|  |  | Skin damage |
|  |  | Heat stress/sunstroke |
|  |  | Dehydration |
|  |  | Headaches |
|  |  |  |
|  | Well-being | Feels (too) warm on skin |
|  |  | Feeling unpleasant |
|  |  |  |
|  | Appearance | Wrinkles |
|  |  | Pigmentation spots |
|  |  | Dry skin |
|  |  |  |
|  | Other | Mosquitos |
|  |  |  |
| ***Positive perceptions*** | Health | Vitamin D |
|  |  | More energy/being more fit |
|  |  | Beneficial for biorhythm |
|  |  | Beneficial for muscles |
|  |  | Beneficial for skin diseases (e.g. psoriasis) |
|  |  | Waking up more easily |
|  |  | Being physically active |
|  |  |  |
|  | Well-being | Feeling good |
|  |  | Feeling happy |
|  |  | Feeling of general well-being |
|  |  | Feeling relaxed |
|  |  | Experiencing freedom |
|  |  | Feeling more at ease |
|  |  | A holiday feeling |
|  |  | Feeling less depressed |
|  |  |  |
|  | Appearance | A tanned skin |
|  |  | Wearing pretty clothes |
|  | *- A tanned skin* |  |
|  | Negative | Degree of coloring |
|  |  | Sunbed-tanned |
|  |  |  |
|  | Positive | Healthy |
|  |  | Pretty |
|  |  | Self-confidence |
|  | Neutral |  |
|  |  |  |
|  | *- Children’s tanned skin* |  |
|  | Negative | Degree of colored skin |
|  |  |  |
|  | Positive | Healthy |
|  |  | Beautiful |
|  |  | Fresh |
|  |  |  |
|  | Neutral |  |
|  |  |  |
|  | Children’s own perception | Funny |
|  |  |  |
|  | *- Social norm* |  |
|  | Positive | Healthy |
|  |  | Beautiful |
|  |  | Compliments |
|  |  |  |
|  | Intentional tanning |  |
|  |  |  |
|  | *-* *Pale skin* |  |
|  | Negative | Unhealthy |
|  |  | Sick |
|  |  | Not beautiful |
|  |  | Spending too little time outdoors |
|  |  |  |
|  | Positive | Beautiful |
|  |  |  |
|  | Neutral | Sensitive skin |
|  |  |  |
|  | *- Other* | Long-lasting days |
|  |  | Flowers |
|  |  | Appealing environment |
|  |  | Friendly and happy people |
|  |  |  |
| **Themes** | **Codes** | **Sub-codes** |
| **2. Sun protection** |  |  |
| ***Parent-for-child*** |  |  |
| *Direct* | Sunscreen application | Prior to sun exposure |
|  |  | Sun Protection Factor (SPF) |
|  |  | Routine use |
|  |  |  |
|  | Seeking shade |  |
|  | Sun avoidance |  |
|  |  |  |
|  | Clothing | Hat |
|  |  | UV-protective clothing |
|  |  | Sunglasses |
|  |  |  |
| *Indirect* | Supportive behavior | Providing information |
|  |  | Helping to remember sun protection |
|  |  | Performing sun protection together |
|  |  | Let children do it themselves |
|  |  | Modeling |
|  |  | Checking |
|  |  | Teaching routine |
|  |  | Providing sun protection materials |
|  |  |  |
| ***Partner-for-child*** | Similar to partner’s sun protection | Consistency |
|  |  | Teamwork |
|  | Different from partner’s sun protection | Organization |
|  |  |  |
| ***Child itself*** | Sunscreen application |  |
|  | Clothing |  |
|  | Seeking shade |  |
|  |  |  |
|  | Child’s perception of own sun protection behavior | Interesting |
|  |  | Pleasant |
|  |  | Cool |
|  |  | Habit |
|  | Differences between boys and girls |  |
|  |  | Girls: younger age, boys: older age |
|  |  |  |
| ***Barriers for sun protection*** | Barriers in the physical environment | Absence of shade |
|  |  | Waterfront far away from shady area |
|  |  |  |
|  | Cognitive barriers | No priority |
|  |  | No preparations/no habitual behavior |
|  |  | Unaware of UV index and weather |
|  |  | Lack of knowledge |
|  |  | Misconceptions |
|  |  | Focus on other activity (e.g. sports) |
|  |  |  |
|  | Economic barriers | Price of sunscreen or clothing |
|  |  |  |
|  | Climate/weather | Wind |
|  |  | Water |
|  |  | Cloudiness |
|  |  |  |
|  | Sunscreen barriers | Type of sunscreen |
|  |  | Warranted SPF |
|  |  | Interval of reapplication |
|  |  | Thickness of sunscreen |
|  |  | Adverse effects of sunscreen |
|  |  | Water resistance |
|  |  | Relying on sunscreen only |
|  |  |  |
|  | Other | Sun protection measures being impractical |
|  |  |  |
| ***Facilitators for sun protection*** | Facilitators in the physical environment | Shady areas |
|  |  | Warning signs |
|  |  | Information provision |
|  |  | Availability of other items (e.g. sunscreen) |
|  |  |  |
|  | Cognitive facilitators | Adequate preparation |
|  |  |  |
|  | Social facilitators | Changed social norm |
|  |  |  |
|  | Political facilitators | Marketing |
|  |  | Innovative strategies (e.g. coloring sunscreen bottle) |
|  |  | Information provision (e.g. via media, at schools, daycare centers, sports clubs) |
|  |  |  |
|  | Other | Skin type |
|  |  | History of sunburn |
|  |  | Skin cancer experience |
|  |  | Experience from other countries |
|  |  |  |
| **Themes** | **Codes** | **Sub-codes** |
| **3. Sunburn** |  |  |
| ***Explicit situations*** | Locations with parents | Swimming pool or beach |
|  |  | While playing |
|  |  | While biking |
|  |  | While in the garden |
|  |  |  |
|  | Locations without parents | At school |
|  |  | At a friend’s place |
|  |  |  |
|  | Location of sunburn | Calves |
|  |  | Legs |
|  |  | Shoulders |
|  |  | Arms |
|  |  | Face |
|  |  | Back |
|  |  |  |
|  | Severity of sunburn |  |
|  |  |  |
|  | Timing | Morning |
|  |  | Afternoon |
|  |  | Spring |
|  |  | Summer |
|  |  |  |
|  | Duration of sun exposure |  |
|  |  |  |
|  | Weather conditions | Temperature |
|  |  | Cloud coverage |
|  |  | Awareness prior to situation |
|  |  | Awareness during situation |
|  |  |  |
|  | Adults present |  |
|  |  |  |
|  | Parents’ feelings/emotions | Shock |
|  |  | Guilt |
|  |  |  |
|  | Awareness of sunburn | During the situation |
|  |  | After the situation |
|  |  |  |
|  | Sun protection methods | None |
|  |  | Sunscreen |
|  |  | Seeking shade |
|  |  | Clothing |
|  |  | Hat and sunglasses |
|  |  | Coloring skin as indicator |
|  |  |  |
| ***Implicit situations*** | Situations with parents | During holidays |
|  |  | While playing |
|  |  | While biking |
|  |  |  |
|  | Situations without parents | While at a sports club |
|  |  | While playing |
|  |  | While at school |
|  |  |  |
|  | Timing | Spring |
|  |  |  |
|  | Duration of sun exposure |  |
|  |  |  |
|  | Weather conditions | Temperature |
|  |  | Cloud coverage |
|  |  | Awareness prior to situation |
|  |  | Awareness during situation |
|  |  |  |
